# Structural and Tribological Assessment of Biomedical 316 Stainless Steel Subjected to Pulsed-Plasma Surface Modification: Comparison of LPBF 3D Printing and Conventional Fabrication

**DOI:** 10.3390/ma14247671

**Published:** 2021-12-12

**Authors:** Yuliia Chabak, Bohdan Efremenko, Ivan Petryshynets, Vasily Efremenko, Angeliki G. Lekatou, Vadym Zurnadzhy, Iurii Bogomol, Victor Fedun, Karol Kovaľ, Tatiana Pastukhova

**Affiliations:** 1 Physics Department, Pryazovskyi State Technical University, 87555 Mariupol, Ukraine; julia.chabak25@gmail.com (Y.C.); bodyaefr@gmail.com (B.E.); vadim.zurnadzhy@gmail.com (V.Z.); fedun276@gmail.com (V.F.); tetianapast@gmail.com (T.P.); 2 Institute of Materials Research, Slovak Academy of Sciences, 04001 Kosice, Slovakia; ipetryshynets@saske.sk (I.P.); kkoval@saske.sk (K.K.); 3 Laboratory of Applied Metallurgy, Department of Materials Science and Engineering, University of Ioannina, 45110 Ioannina, Greece; alekatou@uoi.gr; 4 Institute of Materials Science and Computing, University Research Center of Ioannina (URCI), 45110 Ioannina, Greece; 5 Department of High-Temperature Materials and Powder Metallurgy, National Technical University of Ukraine “Igor Sikorsky Kyiv Polytechnic Institute”, 03056 Kyiv, Ukraine; ubohomol@iff.kpi.ua

**Keywords:** 316L, laser-based powder bed fusion processing, pulsed-plasma treatment, dry sliding wear, SBF-sliding wear, nanoindentation

## Abstract

The structural features and nanoindentation/tribological properties of 316 stainless steel fabricated by conventional rolling and laser-based powder bed fusion (LPBF) were comparatively investigated regarding the effect of surface-pulsed plasma treatment (PPT). PPT was performed using an electrothermal axial plasma accelerator under a discharge voltage of 4.5 kV and a pulse duration of 1 ms. Optical microscopy, scanning electron microscopy, X-ray diffraction, nanoindentation measurements and tribological tests were applied to characterize the alloys. The LPBF steel presented almost the same modulus of elasticity and double the hardness of rolled steel. However, the LPBF steel manifested lower dry-sliding wear resistance compared with its wrought counterpart due to its porous structure and non-metallic inclusions. Conversely, LPBF steel showed three times higher wear resistance under sliding in simulated body fluid (SBF), as compared with wrought steel. PPT led to steel modification through surface melting to a depth of 22–26 μm, which resulted in a fine cellular structure. PPT moderately improved the dry-sliding wear resistance of LPBF steel by fusion of pores on its surface. On the other hand, PPT had almost no effect on the SBF-sliding wear response of the steel. The modification features were analyzed using a computer simulation of plasma-induced heating.

## 1. Introduction

316L is an austenitic stainless steel (SS) that combines high corrosion resistance [1], non-magnetic properties, satisfactory mechanical behavior, good weldability and formability [2,3]. As such, 316L is attractive to many fields, such as the chemical industry, bioengineering, the aerospace industry, etc. [4,5]. During the last decade, additive manufacturing (AM) has often been used for the fabrication of 316L components [6,7,8]. Laser-based powder bed fusion has been proven an effective AM technique for the fabrication of small parts of sophisticated shape, allowing a significant reduction in manufacturing costs and time [1].

The microstructure and properties of LPBF-fabricated 316L steel have been the focus of many researchers. Suryawanshi et al. [9] attributed the microstructure refinement of LPBF 316L to the large thermal gradient in the liquid metal under the laser beam, in contrast to to hot-rolled 316L. The effect of LPBF process parameters on the properties of 3D-printed 316L steel was studied in [9,10]. Analytically, Zhang et al. [10] showed that the optimal laser-scanning angle for attaining good mechanical behavior of 316L is of 30°. Yusuf et al. [11] concluded that high-pressure torsion with more than five rotations is effective for structural refinement and the consequent improvement of the mechanical properties of LPBF 316L. Several works have been dedicated to the effect of post-heat treatment on the properties of LPBF 316L [12,13,14,15]. Garthe et al. [14] reported that a stress-relief post-heat treatment improves the low cycle fatigue behavior of LPBF 316L. Other studies claim that the post-heat treatment of LPBF 316L does not affect its phase state and crystallographic orientation [12,13].

LPBF 316L, as a biological implant, undergoes complex loading inside the human body, which may lead to frictional damage of the involved parts. Despite indisputable advantages, an austenitic SS is characterized by a low hardness and poor tribological behavior [16,17] resulting in surface damage under sliding. This is undesirable for a human-body implant application since the release of wear debris and Ni/Cr toxic ions may cause inflammatory and anaphylactic reactions [18,19]. Therefore, the wear performance of 316L should be improved in order to ensure a safe application of the implant and prolong its surface life. For this purpose, different techniques of surface treatment have been proposed [20,21,22,23]. Rosenkranz et al. [24] claim that the synergy of surface texturing and solid lubricants may decrease both wear and friction. Duriagina et al. [25,26] achieved an improved wear resistance of 316L SS when they applied surface-laser Nb-Fe-Ni-B-C alloying under a supervised learning technique. García-León et al. [27] employed a powder-pack boriding process to generate a boride-based layer on the surface of 316L, and they achieved a higher dry sliding wear resistance compared with that of the 316L substrate. An original approach was described by Li et al. [28], who improved the corrosion resistance of LPBF 316L steel by adding 10 vol.% of Co-Cr-Mo-W powder to the powder bed of 316L. As follows from several works [29,30,31], different plasma-related processes may be adopted to treat SS. Kovaci and Secer [29] increased the wear resistance of 316L SS by laser texturing and further plasma nitriding. The same approach was followed by Wang et al. [3] and Aliofkhazraei et al. [30]. The latter effort increased the SS surface hardness and corrosion resistance by plasma electrolytic nitrocarburizing.

Pulsed-plasma treatment (PPT) aims at surface hardening through the structural or chemical modification of the near-surface layer [31,32,33]. A high heating/cooling rate induced by collision with plasma flux results in surface-structure modification and refinement. PPT was previously applied by the authors in order to achieve surface hardening of steels [33,34], grey cast iron [35] and Ti-based alloys [36]. PPT has also been used for coating deposition [37,38] and high-Cr cast-iron structure refinement [39]. The wear performance of LPBF 316L steel has been investigated by many researchers [2,3,27,40,41,42,43]. However, the aforementioned studies rarely focused on the post-treatment of the AM surface. Based on the above, the present research aims to study of the effect of surface-pulsed plasma treatment on the structure and wear behavior of LPBF 316L stainless steel, from the viewpoint of comparison with conventionally manufactured and pulsed-plasma-treated 316L. 

## 2. Materials and Methods

316L steel specimens were fabricated by LPBF using a 3D printer (ProX DMP 320 by 3D Systems, Rock Hill, SC, USA) equipped with a fiber laser and a working camera 275 × 275 × 420 (mm) in size. The LPBF parameters are listed in Table 1 (standard for this type of material). EOS 316L metal powder (manufactured by Electro Optical Systems, Turku, Finland) was used as source material, with a chemical composition of (wt.%): 16.8 Cr, 2.25 Mo, 10.8 Ni, 0.48 Si, 1.18 Mn, 0.030 P, 0.030 C, 0.006 S, Fe—base. The average powder-particle size was not greater than 63 μm, and the density of the steel was 7.98 g/cm^3^. The LPBF-fabricated specimen size was 5 × 10 × 20 (mm), with an average surface roughness of R_a_ = 3.086 μm and R_z_ = 6.535 μm.

The post-process (pulsed-plasma treatment) was performed using an electrothermal axial plasma accelerator (EAPA) built at Pryazovskyi State Technical University (Mariupol, Ukraine) and described in detail in [44,45]. Briefly, EAPA is a paper-reinforced bakelite tube of 430 mm length, 8 mm inner diameter and 17 mm wall thickness. An anode (a steel flange) and a cathode (tungsten rod) were positioned inside the EAPA axial chamber at a distance of 60 mm in order to switch on a high-voltage arc discharge. The EAPA was connected to a capacitive energy storage device of 1.5 mF capacity. Arc discharge with a current of ~15–18 kA and a voltage of ~4.0–4.5 kV during 0.5–1.0 ms leads to a rapid release of about 10 kJ due to evaporation/melting of the inner walls and electrodes, causing a pressure increase of up to 100–150 atm. This enables the formation of a plasma flux and its fast injection out of the EAPA chamber [44]. PPT was performed under the working parameters presented in Table 1. These parameters were selected based on previous experience [35,37,38] in order to modify the steel specimen through superficial melting.

LPBF 316L steel was compared with conventionally manufactured (rolled) 316T steel. The latter was provided by Outokumpu, Helsinki, Finland, as an 8 mm thick rolled sheet of chemical composition (wt.%): Fe (base), 17.11 Cr, 2.07 Mo, 10.66 Ni, 0.44 Si, 1.34 Mn, 0.36 Ti, 0.035 P, 0.048 C, 0.001 S, Fe. 316T grade has a chemical composition very close to that of 316L, except for a slightly higher carbon content and titanium addition needed to bond the carbon excess into the carbide phase.

The microstructure of transversely cross-sectioned specimens was prepared according to standard procedure of mirror polishing by SiC sandpapers and alumina aqueous solutions with further etching by Aqua Regia reagent (a mixture of HNO_3_ and HCl at a ratio of 1:3) and examined by optical microscopy (OM) and scanning electron microscopy/energy-dispersive x-ray spectroscopy (SEM/EDX) using the “GX71” optical microscope (Olympus, Tokyo, Japan) and the “JSM-7000F” scanning electron microspore (JEOL, Tokyo, Japan) equipped with the EDX “INCAx-sight” detector (Oxford Instruments, Abingdon, UK). Phase identification was performed by X-Ray diffraction using the “X’Pert PRO” diffractometer (PANalytical, Malvern, UK) with Cu-Ka radiation.

The determination of micromechanical properties (microhardness, elastic modulus) was performed using the “Nano Indenter G200” device (Agilent Technologies, Santa Clara, CA, USA) at a loading speed of 10 nm⋅s^−1^.

Tribological behavior was evaluated at room temperature by the “ball-on-disc” technique using the “Micron-tribo” tribometer (Micron-System, Kyiv, Ukraine) as follows: The specimen (disc) contacted a ball under the normal load of 5 N, making reciprocating movements, with a sliding speed of 7.0 mm⋅s^−1^. The total sliding distance was 5.25 m (1500 strokes, each 3.5 mm long) for dry sliding and 17.50 m (5000 strokes, each 3.5 mm long) for wet sliding. A corundum ball of 3 mm diameter was employed as a counter body. Wet sliding was performed in a simulated body fluid (SBF) with the following ion concentration (mM/L): Na^+^ = 145.0, K^+^ = 4.0, Mg^2+^ = 1.0, Ca^2+^ = 2.5, Cl^−^ = 127.0, HCO_3_^−^ = 24.0 [46].

Since the pulsed-plasma treatment caused an increase in the surface roughness of the steel specimens, prior to the wear test, the plasma-treated surface was gently polished in order to attain a sufficient smoothness (Ra = 0.07 μm, Rz = 0.40 μm), taking special care not to remove the plasma-induced layer. The wear track was profiled by the “Micron-beta” profilometer (Micron-System, Kyiv, Ukraine) to build a 3D model. The sliding behavior was evaluated by the mean coefficient of friction (CoF) and ΔV (volume difference). The coefficient of friction was determined by the tribometer, recording one value for each stroke. Then, the mean CoF value was calculated by averaging the current values for all strokes. The volume difference was determined as ΔV = VL–VI, where VL (volume loss) is the wear-track-deepening volume, and VI (volume increase) is the volume of the protrusions at both sides of the wear track. VI and VL values were extracted from the wear-track profile relative to the profile baseline. ΔV value was calculated by the profilometer “Micron-beta” (Micron-System, Kyiv, Ukraine) for a wear track of 785 μm length [38]. ΔV value refers to the specimen’s wear, meaning the material removed from the surface during the test. Each ΔV value is the average of three test repetitions.

## 3. Results

### 3.1. As-Received Microstructure and Nanoindentation Response

**Figure 1:**
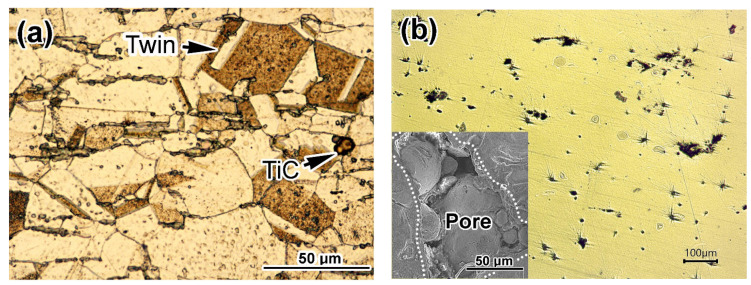

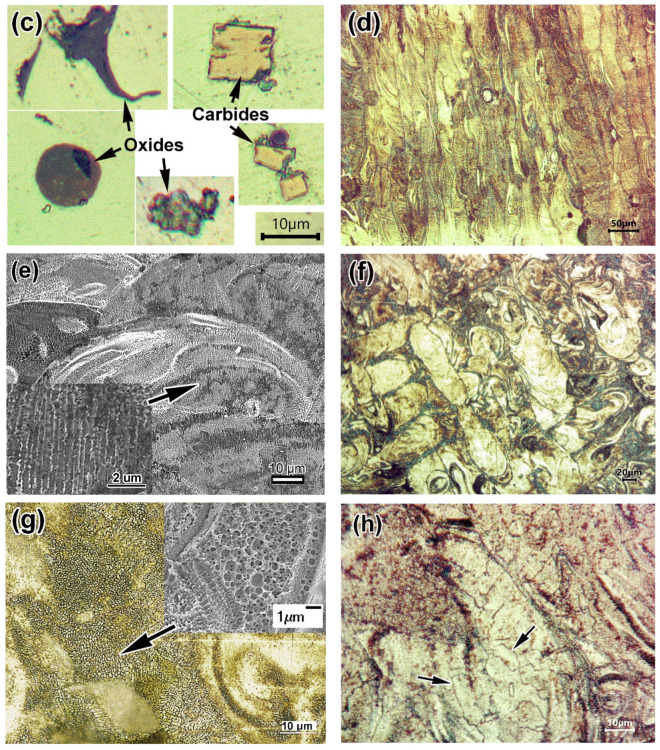
Microstructural features of the as-received specimens: (**a**) plate 316T, (**b**) non-etched LPBF 316L, (**c**–**h**) etched LPBF 316L.

The microstructure of the plate 316T is presented in Figure 1a. It has the typical appearance of a single-phase austenitic alloy, consisting of polyhedral grains of gamma solid solution with twins, which are characteristic of metals with an FCC lattice (Figure 1a). The size of the austenitic grains varies from 9 μm to 41 μm, with an average value of 20.7 ± 10.1 μm. Intergranular faceted inclusions of titanium carbide with a maximum size of ~5 μm are occasionally observed in the structure.

**Figure 2:**
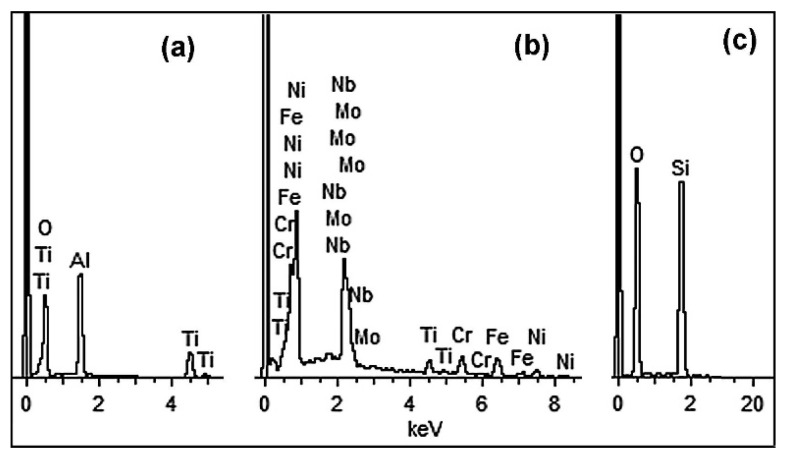
EDXspectra of (**a**) oxide (Ti,Al)O_2_, (**b**) carbide (Nb,Mo,Ti)C and (**c**) oxide SiO_2_ in LPBF 316L.

The low-magnification micrograph of the non-etched LPBF 316L specimen in Figure 1b reveals a rather incompact structure containing pores (decades of micrometers in size—see inset of Figure 1b) and non-metallic inclusions, which have caused the formation of comet-tails. The total area fraction of pores is 1.52%. Figure 1c presents the inclusions in a higher magnification, before etching. EDX analysis allowed for the identification of the cubic faceted precipitates (with the morphologic features of MC carbide) as (Nb,Mo,Ti)C (the chemical composition is listed in Table 2, and the corresponding EDX-spectra are shown in Figure 2). The presence of Cr, Fe and Ni in the carbide can be explained by the EDX response of the matrix around the precipitate trapped within the detector area. The globular and scale-like inclusions were identified as (Ti,Al)O_2_ and SiO_2_. The results presented in Table 1 should be considered as semi-quantitative since carbon was excluded from the analysis because of the high EDX sensitivity to carbon contamination.

**Figure 3:**
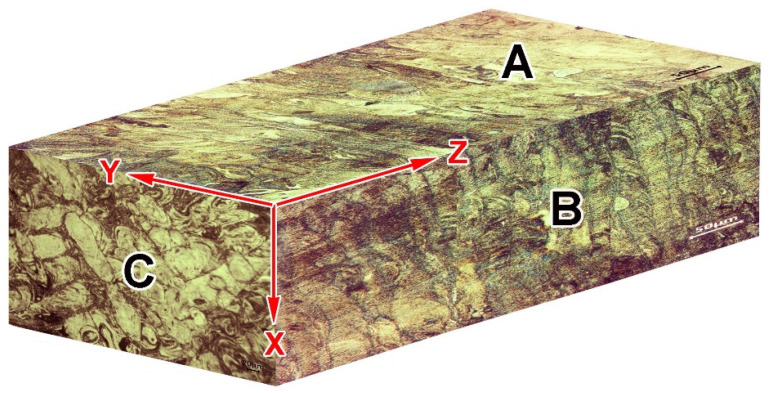
Microstructure distribution in the LPBF-printed specimen (Z is the axis of layering during printing).

Figure 1d,f show that after etching, the structure of LPBF 316L steel appears heterogeneous, while the structural pattern depends on the specimen-sectioning direction. More analytically, Figure 3 shows the scheme of microstructure distribution regarding the printing axes. On surfaces A and B, the structure has a typical “fish-scale” pattern [47] resulting from layer-by-layer material consolidation. This pattern is illustrated in Figure 1d. The width of the layers (which are actually chains of the individual “scales”) vary in the range of 14–45 μm, with an average value of 29.2 ± 5.1 μm. The layers are positioned perpendicular to the long side of the specimen, which coincides with the *Z*-axis of printing. A “fish-scale” is shown in Figure 1d,e; the hemispherical shape with convexity in the Z direction (Figure 1e) reflects the shape of a liquid-powder particle on the substrate surface melted under the laser beam. The body of the “fish-scale” consists of columnar dendrites of 0.3–0.5 μm width in cross-section oriented mostly parallel to the *Z*-axis (inset of Figure 1e). In contrast, in the XY plane (side C), which served as the basis for layering in LPBF printing, the structure has a pattern of intermittent, “intertwined” rows of elongated elements 25–50 μm wide and 120–170 μm long positioned along two mutually perpendicular directions, reminiscent of the location of the threads in the fabric (Figure 1f). The longish dendrites in Figure 1f are enveloped by dark contrast zones. The different contrast of the separate areas reflects their resistance to etching, owing to variations in structure and chemical composition. Figure 1h shows that the light areas have a relatively smooth surface, forming grains (the grain boundaries of which are noted by arrows in Figure 1h). At a higher magnification, the dark areas present a cellular pattern with a cell diameter less than 1 μm (see inset of Figure 1g). The cells are actually the cross-sectional projection of the columnar dendrites seen in Figure 1e. The structural elements were formed by molten powder particles (in plane), and their length and location in the “woven” pattern correspond to the movement of the laser beam during the LPBF printing.

**Figure 4:**
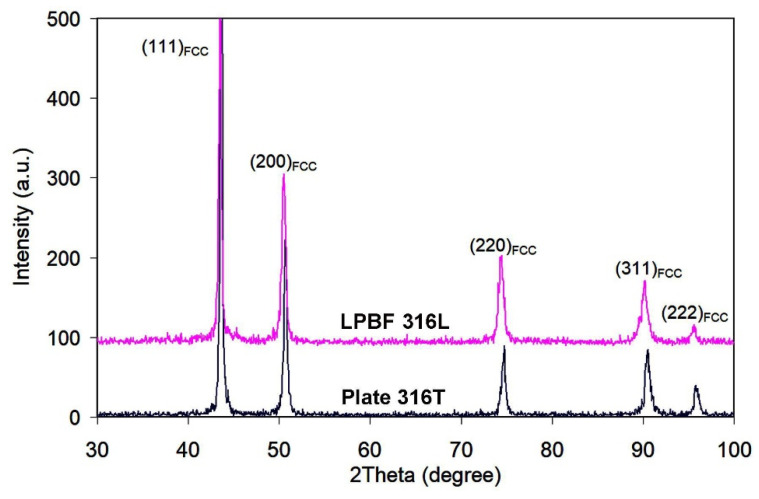
XRD patterns of as-received LPBF 316L and plate 316T specimens.

The qualitative phase composition of the as-received plate and LPBF specimens was investigated by X-ray diffraction. Only the (111), (200), (220), (311) and (222) diffraction maxima belonging to gamma-Fe are discerned in both diffractograms of Figure 4. Thus, both plate 316T and LPBF 316L specimens present a fully austenitic (FCC lattice) structure. Other phases, such as NbC, are not detected, indicating an insignificant content. Despite the identical set of diffraction maxima for the two manufacturing technologies, XRD-patterns differ in diffraction maxima width. The values of FWHM (full width at half maximum) were calculated for the most intensive peaks, (111) and (200). The FWHM values for the plate 316T specimen are: FWHM_(111)_ = 0.30 degree; FWHM_(200)_ = 0.35 degree. The FWHM values for the LPBF 316L specimen are: FWHM_(111)_ = 0.35 degree; FWHM_(200)_ = 0.50 degree. Therefore, LPBF 316L presents broader XRD peaks than plate 316T, indicating a greater crystal lattice distortion, affected by micro-stresses and lattice defects due to the rapid solidification of the molten particles and the cyclic reheating by repetitive laser-beam scanning.

**Figure 5:**
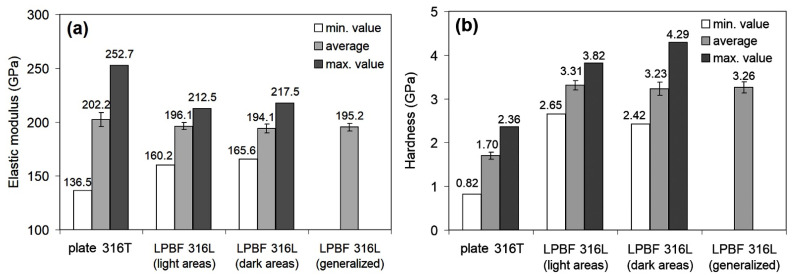
Nanoindentation results of the as-received plate 316T and LPBF 316L; (**a**) elastic modulus; (**b**) hardness.

Micromechanical properties of the specimens were determined by nanoindentation according to a 10 × 10 matrix with a step of 50 μm between the prints in two directions. The maximum indenter depth under indentation was 2000 nm. The obtained values of elastic modulus (E) and hardness (H) are given in Figure 5.

**Figure 6:**
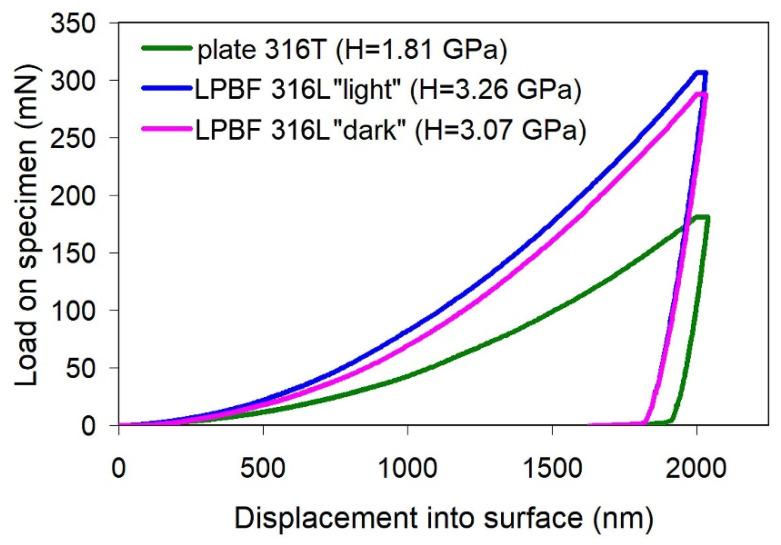
Representative load/displacement curves of as-received plate 316T and as-received LPBF 316L specimens.

The E values for plate 316T scatter from 136.45 GPa to 252.70 GPa, with an average value of 202.21 ± 6.51 GPa. Accordingly, the hardness values are in the range of 0.823–2.36 GPa, with an average value of 1.704 ± 0.08 GPa. A typical load/displacement curve for plate 316T specimen is presented in Figure 6.

Considering the structural heterogeneity of the LPBF 316L specimens, nanoindentation was performed on side C in such a way that the measurements in the “light” and “dark” structural areas were processed separately. The data obtained (Figure 5) show that both the scatter and the mean values of elastic modulus (E), as well as hardness, are similar for both areas. After generalizing the data for the different contrast areas, the mean values of E (195.20 ± 3.68 GPa) and hardness (3.26 ± 0.13 GPa) were obtained for the LPBF 316L specimens.

Figure 5 shows that plate 316T and LPBF 316L specimens demonstrate almost the same level of elastic modulus, with a slight (by ~7 GPa) advantage of the plate specimens. Notably, the LPBF 316L specimens show a much lower scatter and confidence interval of E, despite the significant heterogeneity of their structure. Furthermore, the LPBF 316L specimens are almost twice as hard as the plate specimens (3.26 GP and 1.70 GPa, respectively), with a slightly higher scatter in values. The relatively high scatter of the hardness values of the LPBF-printed steel is compatible with the relatively high lattice distortion detected by X-ray diffraction (Figure 4). Finally, it is concluded that the LPBF-printed 316L steel is, in general, not inferior to plate 316T steel as regards micro-mechanical properties.

### 3.2. Plasma Heating Simulation

In this work, pulsed-plasma treatment was applied to the investigated stainless steels in order to modify their surface through plasma flux heating. Prior to PPT, a computer simulation was carried out to predict the results and confirm the processing parameters. The simulation was fulfilled according to the approach outlined in detail in a previous effort by the authors [38]. Calculations were performed based on the approach of short-term heat sources, q_(t, x)_, evenly distributed over the treated surface. The applied PPT regime (i.e., voltage of arc discharge of 4.5 kV, arc duration of 1.0 ms) corresponded to q_(t, x)_: 6 × 10^8^ Wt⋅m^−2^. The calculations took into account the known values of temperature dependences of critical thermophysical parameters (density, specific heat capacity, thermal conductivity), as well as the solidus temperature (t_Sol_) of 1675 K for steel 316L, adopted from [48,49,50].

**Figure 7:**
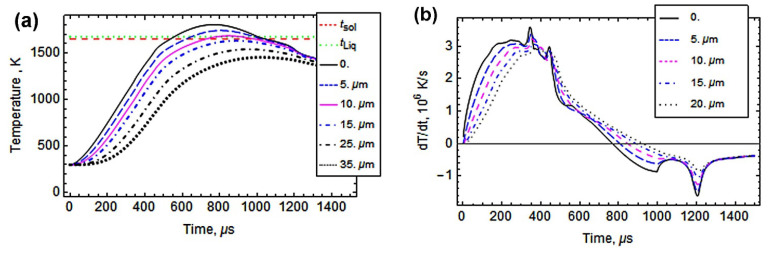

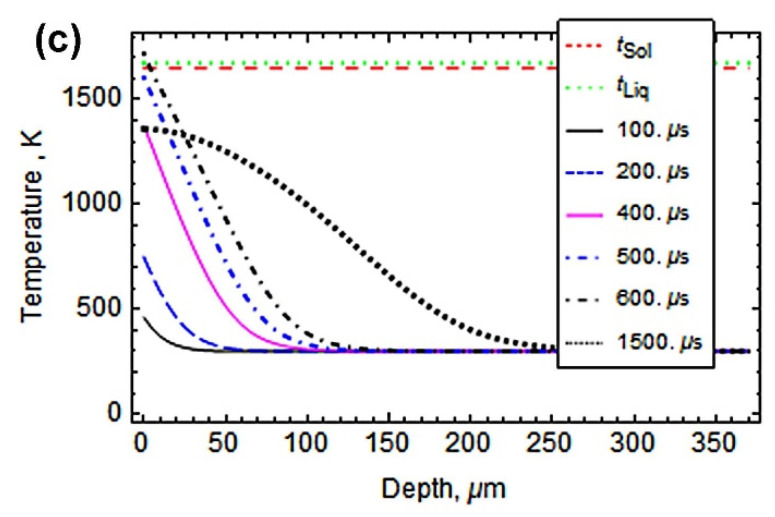
Temperature field dynamics in the specimen of steel 316L after contacting with the plasma pulse: (**a**) layer-by-layer temperature change, (**b**) layer-by-layer change in heating/cooling velocity, (**c**) depth-wise temperature change over time.

The simulation (results presented in Figure 7) allows for the analysis of the alteration of temperature in the surface layers of 316L steel when the specimen collides with a plasma pulse. Figure 7a shows that under plasma heating, the surface temperature peaks at 1820 K (after 750 μs), which is above the solidus temperature. The temperature of the layer at a depth of 15 μm from the surface peaks at t_sol_, meaning that melting of the surface proceeded to a depth of 15 μm. Since heating lasts less than 1000 μs, quite a high heating velocity should be attained, reaching a maximum of 3.6 × 10^6^ K⋅s^−1^ on the surface (in 350 μs) and a maximum of 2.9 × 10^6^ K⋅s^−1^ at a depth of 20 μm from the surface (in 450 μs), as shown in Figure 7b. After attaining the maximum temperatures, cooling starts due to heat removal into the specimen’s bulk. After about 1000 μs, the surface temperature decreases to t_Sol_ (Figure 7a), implying crystallization of the molten layer. Within 1200 μs after the start of plasma-flow exposure, the cooling velocity reaches 1.6 × 10^6^ K⋅s^−1^ at the surface and 0.8 × 10^6^ K⋅s^−1^ at a depth of 20 μm (Figure 7b). Such high cooling velocity may lead to “freezing” of the molten layer, with formation of a superfine structure. Thus, the simulation confirms that PPT with the selected regime parameters could be applied for surface modification through surface melting up to a depth of 15 μm, followed by ultrafast crystallization.

### 3.3. Microstructure and Properties after Pulsed-Plasma Treatment

**Figure 8:**
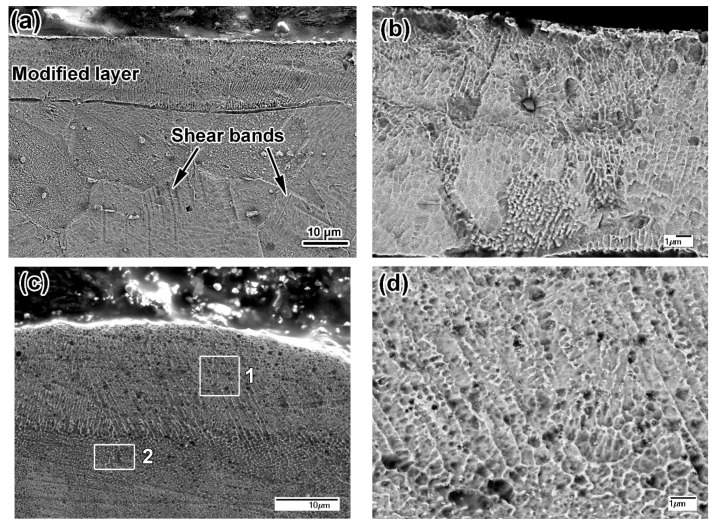

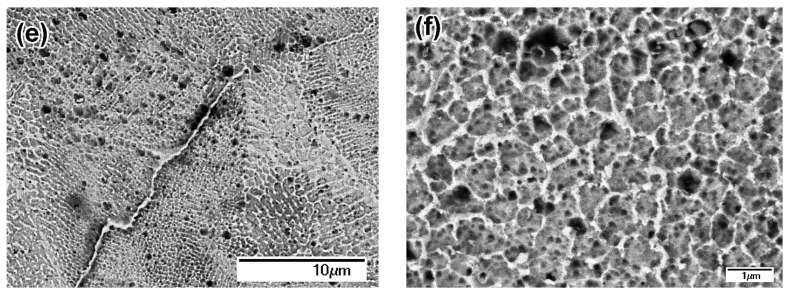
SEM images of pulsed-plasma-treated specimens: (**a**,**b**) plate 316T, (**c**–**f**) LPBF 316L ((**a**,**c**,**e**,**f**)—SEI; (**b**,**d**)—BSE). (**d**) refers to square 1 in (**c**). (**e**,**f**) refer to rectangle 2 in (**c**).

The microstructure of the plasma-modified specimens is illustrated in Figure 8. As follows from Figure 8a, PPT of plate316T resulted in a modified layer of 13–18 μm thickness separated from the bulk by a distinct boundary. Below the boundary, shear bands can be discerned inside the austenitic grains to a depth of 50 μm; they were caused by thermal deformation during high-velocity plasma heating. The modified layer has a columnar structure (Figure 8b), characteristic of directionally crystallized melt, proving that melting occurred under plasma heating.

The same pattern is observed in the modified layer of the pulsed-plasma-treated surface of LPBF 316L (Figure 8c). The PPT layers appear denser (without the large pores seen in Figure 1b), as compared to the as-printed state. The total depth of the modified structure is 22–26 μm, with a smooth transition to the bulk structure and no distinct boundary. Figure 8c shows that the modified layer consists of two sublayers. The details of the structure of the upper sublayer are shown in Figure 8d, which is an enlarged image of the area inside square 1 in Figure 8c. The upper sublayer of 15–18 μm thickness is composed of bunches of columnar coarse dendrites (0.3–0.7 μm in cross-section) perpendicular to the free surface. The structure of the low sublayer is illustrated in Figure 8e,f, which are enlarged images of the area inside rectangle 2 in Figure 8c. The low sublayer exhibits less pronounced columnarity and more equiaxed cells; moreover, a “net” along the grain boundaries is seen within this layer.

**Figure 9:**
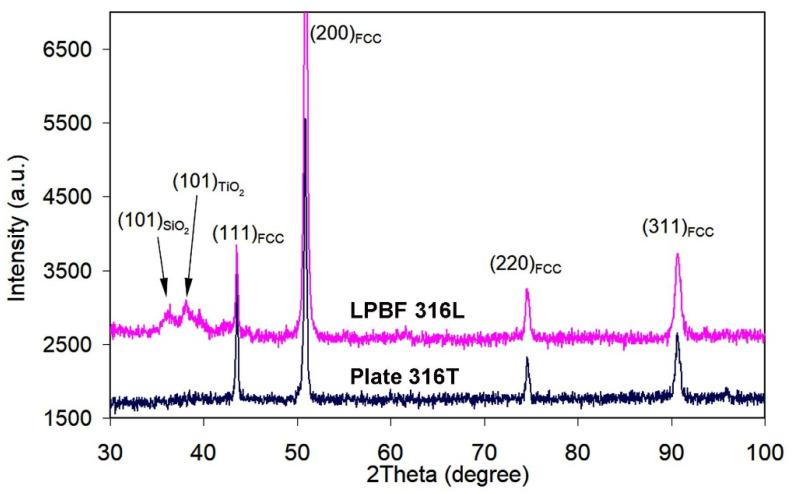
XRD patterns of pulsed-plasma-treated specimens.

The X-ray diffractograms of the plasma-modified specimens are presented in Figure 9. The XRD patterns of both specimens consist of austenite peaks, while the XRD pattern of LPBF 316L additionally shows blurred maxima at a low angle, which may be attributed to SiO_2_ and TiO_2_. FWHM for plate316T: FWHM_(111)_ = 0.23 degree; FWHM_(200)_ = 0.30 degree; FWHM for LPBF 316L: FWHM_(111)_ = 0.24 degree; FWHM_(200)_ = 0.40 degree. Hence, in this case, the FWHM values for both specimens are lower than those for the as-received state. Conversely to the XRD patterns of the as-received steels (Figure 4), in the diffractograms of both PPT steels, the diffraction peak (200)_FCC_ exhibits much higher intensity than that of peak (111)_FCC_ (The (111)_FCC_ peak is the strongest XRD peak of γ-Fe.). This “reversal” signifies that the specimens acquired a specific texture of (200)_FCC_ under the plasma heating/shockwave action on the steel surface.

**Figure 10:**
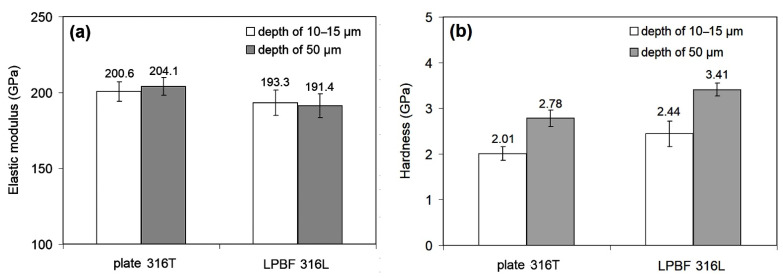
Nanoindentation response of the pulsed-plasma-modified specimens at different depth: (**a**) elastic modulus, (**b**) hardness.

Nanoindentation of the plasma-modified specimens was conducted on cross-sections along two lines parallel to the surface: the first line of imprints lies within a modified layer at a depth of 10–15 μm from the free surface; the second lies at a depth of ~50 μm (within the underlayer) (Figure 10). A comparison of data from Figure 5 and Figure 10 shows that PPT had almost no effect on the modulus of elasticity; on the other hand, PPT affected the hardness of both steels. Specifically, the mean hardness of plate 316T increased from 1.70 ± 0.08 GPa (in the as-received state) to 2.01 ± 0.15 GPa in the modified layer and to 2.44 ± 0.28 GPa in the underlayer. In the case of LPBF 316L, PPT resulted in some decrease in the mean hardness of the modified layer, from 3.26 ± 0.13 GPa to 2.78 ± 0.18 GPa, whereas the underlayer retained a bulk hardness of 3.41 ± 0.14 GPa.

**Figure 11:**
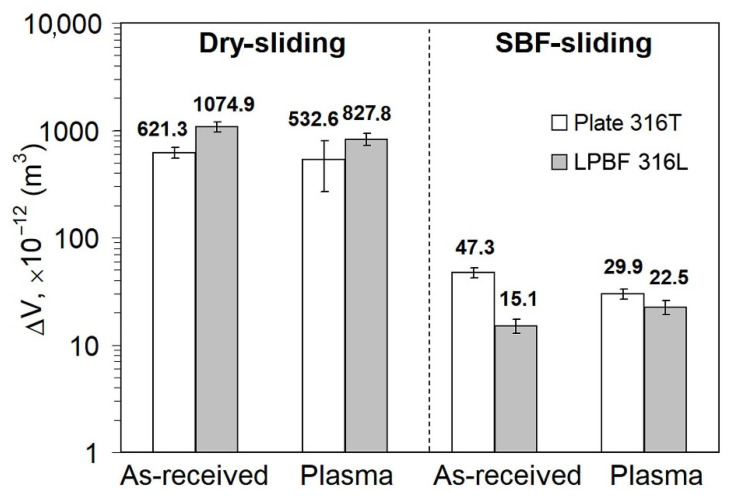
Wear of a plate 316T and LPBF 316L after sliding tests.

**Figure 12:**
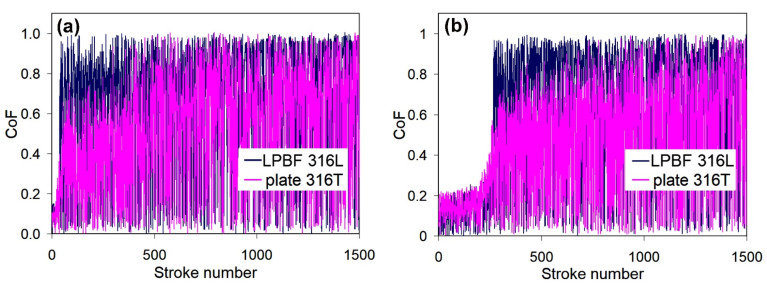
Variations of CoF for the specimens during dry sliding wear: (**a**) as-received and (**b**) plasma-treated stainless steels.

**Figure 13:**
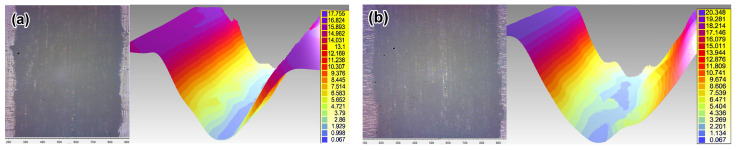

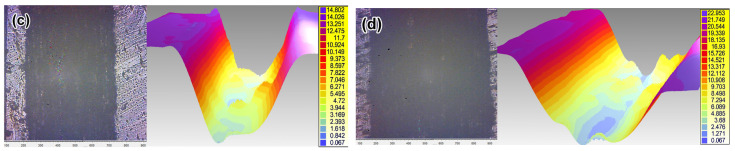
Wear tracks in plane and their 3D images after dry sliding: (**a**,**b**) as-received specimens, (**c**,**d**) plasma-treated specimens ((**a**,**c**): plate 316T; (**b**,**d**): LPBF 316L) (the bar values are given in μm).

**Figure 14:**
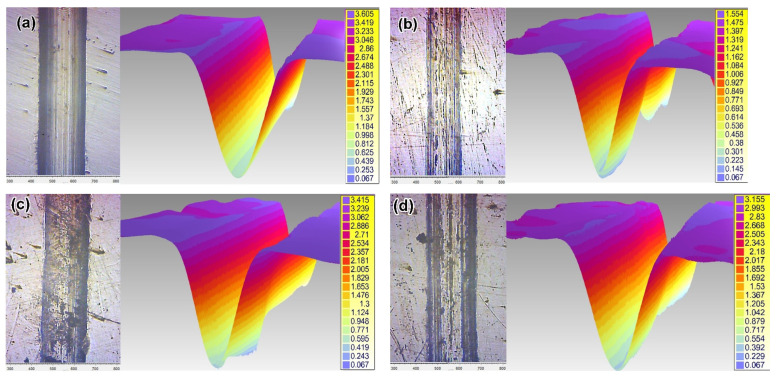
Wear tracks in plane and their 3D images after SBF sliding: (**a**,**b**) as-received specimens, (**c**,**d**) plasma-treated specimens, ((**a**,**c**): plate 316T; (**b**,**d**): LPBF 316L) (the bar values are given in μm).

**Figure 15:**
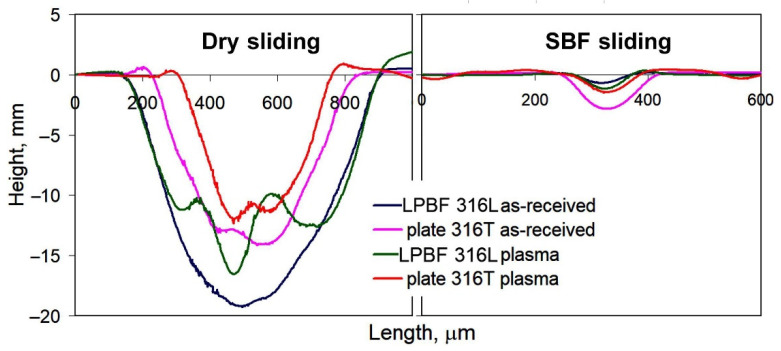
Profiles of wear tracks.

**Figure 16:**
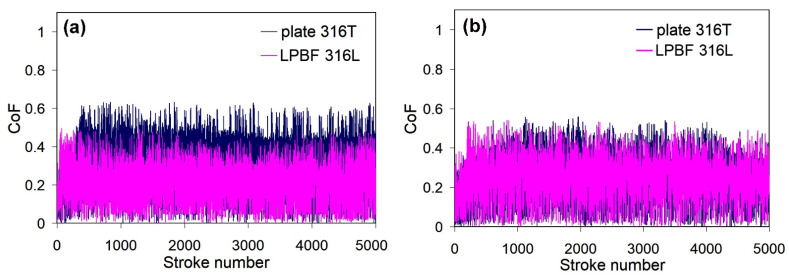
Variation of CoF of the specimens during SBF sliding: (**a**) as-received and (**b**) plasma-treated states.

The tribological behavior of the studied specimens is manifested in Figure 11, Figure 12, Figure 13, Figure 14, Figure 15 and Figure 16.

**Regarding the effect of LPBF:** As seen in Figure 11, as-received LPB F 316L exhibits much higher dry-sliding wear (by 73%) than as-received plate 316T, despite the relatively high hardness of LPBF 316L. Figure 12a shows that dry sliding of both specimens is characterized by intensive friction, with a high friction coefficient; wide scatter bands stretch from CoF = 0 to CoF = 1.0 (the scatter bands of both specimens overlap one another). At the same time, the friction coefficient of plate 316T is somewhat lower than that of LPBF 316L (CoF mean values of 0.52 and 0.68, respectively). The wear tracks after dry sliding appear rather wide (630–750 μm) and deep (16–19 μm) for both steels, as shown in Figure 13a,b.

**Regarding the effect of PPT:** Figure 11 (left) shows that pulsed-plasma treatment resulted in a small improvement in the dry-sliding wear resistance of both specimens; ΔV values decreased by 14% for plate 316T and by 22% for LPBF 316L, i.e., plate specimens have retained their advantage over LPBF-printed specimens, although the differences in the ΔV values between the steels were mitigated. Figure 11 also shows that pulsed-plasma modification enhanced the wear resistance of plate 316T in SBF by 37%. At the same time, PPT led to an increase in the ΔV value of LPBF 316L during SBF sliding, from 15.1 × 10^−12^ m^−3^ (as-received state) to 22.5 × 10^−12^ m^−3^, which is an increase of ~33%.

Figure 12 shows that the CoF scatter of PPT LPBF 316L is as large as that of as-received LPBF 316L (mean value of 0.62). On the other hand, the CoF scatter for PPT plate 316T appears to have decreased (mean value of 0.46) in comparison with the as-received plate 316T.

Figure 16 shows that PPT did not significantly altered the friction response of the LPBF 316L specimen in SBF. The obtained CoF mean value of PPT LPBF 316L (0.25) is slightly higher than that of as-received LPBF 316L (0.21) in SBF. On the other hand, PPT resulted in lower CoF values and scatter of plate 316T compared to as-received plate 316T.

**Regarding the effect of wear media:** Figure 11 shows that wet sliding in SBF led to a significant decrease in the wear of both steels and both the as-received and plasma-treated states, as compared to the dry-sliding process. As-received plate 316T showed a cumulative volume loss of ΔV = 47.3 × 10^−12^ m^3^ during SBF sliding, which is 13 times less than the volume loss during dry sliding (ΔV = 621.3 × 10^−12^ m^3^). The wear of as-received LPBF 316L dropped to an even greater extent; namely, the volume loss of ΔV = 15 × 10^−12^ m^−3^ during SBF sliding of as-received LPBF 316L is 71 times less than that of as-received LPBF 316L ΔV = 1074.9 × 10^−12^ m^3^ during dry sliding. This can also be seen from the comparison of the wear-track profiles in the dry-sliding mode and the SBF sliding mode in Figure 13, Figure 14 and Figure 15: Both the width and the depth of the wet sliding wear tracks appear markedly smaller compared to the dry-sliding wear tracks.

Moreover, Figure 14a,b show that under SBF sliding, as-printed LPBF 316L had a 3-fold advantage over plate 316T, exhibiting narrower and shallower wear tracks, as compared to plate 316T.

The wear decrease under SBF sliding was caused by liquid lubrication of the counter surfaces. Accordingly, a decrease in the scatter and mean value of the friction coefficient was observed for the as-received steels, as shown in Figure 16a. Specifically, the mean CoF value dropped from 0.68 in the dry-sliding mode to 0.21 in the SBF sliding mode for as-received LPBF 316L and from 0.52 in the dry sliding mode to 0.33 in the SBF-sliding mode for plate 316T. A similar trend is observed for the PPT steels (Figure 16b). Specifically, the mean CoF value dropped from 0.62 in the dry-sliding mode to 0.25 in the SBF-sliding mode for as-received LPBF 316L and from 0.46 in the dry sliding mode to 0.26 in the SBF-sliding mode for plate 316T.

Thus, under SBF-sliding, the wear of the specimens is closely associated with their friction behavior.

## 4. Discussion

According to a well-adopted engineering approach, the mechanical behavior of a material should be analyzed based on its structural features. As shown above, the structure of LPBF-printed 316L greatly differs from that of plate 316T. In fact, the LPBF specimen is an agglomerate of cast fine cellular micro-areas of a “fish-scale”-like elliptical shape fused to one another (Figure 1d,e). Each area was formed by the melting of powder particles under a laser beam. After melting, the metal drop acquired a hemispherical shape (Figure 1e) under surface-tension force. This shape was frozen by rapid crystallization through the formation of a fine cellular structure toward the free surface, which is characteristic of LPBF metals [51,52]. According to Zhong et al. [53], the morphological features of this structure are mostly controlled by the temperature gradient (G) within the liquid drop, as well as the solidification rate (R). The XRD study in the present work shows that LPBF-printed 316L acquired an austenitic structure, although the grain boundaries were not revealed by standard etching. (According to Bertoli et al. [54], individual grains are stretched along the Z direction for hundreds of microns, spanning across several printed layers).

Rapid solidification caused thermal stress and deformation of the cellular structure, leading to lattice distortion, as revealed by widening XRD peaks (Figure 4). Structural refinement (through fine cell formation) and residual micro-stress resulted in double hardness of LPBF 316L, as compared to plate 316T; the latter has an equiaxed austenitic structure with polygonal grains. Despite a higher hardness, LPBF 316L shows lower dry sliding wear resistance than plate 316T. The most likely reasons for such wear behavior are the inhomogeneity of the LPBF structure and the imperfection of the LPBF structure manifested in the pores and coarse, non-metallic inclusions, such as oxides, carbides and silicates. The latter act as stress concentrators, promoting surface deterioration and facilitating wear-debris detachment. The presence of Ti-rich inclusions (carbides and oxides) is unexpected since titanium is not a constituent of 316L steel grade (ASTM A240), and it should not be present in the studied 3D-printed specimens (according to the supplier’s information). Presumably, the occurrence of titanium was caused by accidental contamination of the powder or LPBF chamber. Due to the titanium contamination, the LPBF 316L composition is very close to that of plate 316T; thus, both steels are fully comparable.

Pulsed-plasma heating resulted in modification of the surface layers of both steels due to melting. This is most evident for plate 316T, where the structure was altered from that of polygonal grains to a cellular pattern (Figure 8b). Accordingly, the hardness of the melted layer increased relative to the non-modified bulk structure. At the same time, the surface-layer hardness is lower than the hardness of the underlayer (at 50 μm depth) by 0.43 GPa (Figure 10b), indicating that the underlayer experienced strain hardening under thermal stresses (proved by shear-band emergence in Figure 8a). A similar situation is observed for the LPBF 316L specimen; the hardness of the PPT-modified modified (melted) layer is rather inferior to that of the as-printed structure, by 0.48 GPa (Figure 5 and Figure 10). This is despite the fact that the plasma-melted layer and LPBF-printed bulk have the same cellular structure pattern, with close cell size. The similar structure allows for the presumption that the relatively low hardness of the modified layer has an instrumental cause, owing to the proximity of the nanoindenter imprints (depth of 10–15 μm) to the free surface.

Pulsed-plasma treatment led to the emergence of a (200)-plane oriented texture in both plate 316T and LPBF 316L specimens (Figure 9). Additionally, the XRD pattern of the PPT LPBF 316L specimen includes peaks of Ti-rich and Si-rich oxide phases; these phases were not detected in the other cases (including the plasma-treated 316T specimen). Hence, the most plausible reason for detection of such oxide peaks is the contamination of 3D-printed 316L specimens by non-metallic inclusions (Figure 1). The results of the EDX analysis of as-received LPBF 316L (Table 1, Figure 2) confirm that these inclusions are oxides (Ti,Al)O_2_ and SiO_2_. The melting temperatures of these oxides (1999 ± 10 K for SiO_2_ and 2165 ± 30 K for TiO_2_ [55]) exceed the melting point of 316L steel and the maximum temperature of plasma heating (Figure 7a). Thus, it is postulated that the oxide inclusions were not melted in 316L under plasma heating. They might float up in the molten layer (due to their lower density) or be pushed to the surface by growing columnar crystals, leading to the purification of the plasma-modified layer. The increased occurrence of the oxides in very close vicinity of the surface allowed them to be detected by X-ray diffraction.

Tribological testing demonstrated that PPT led to a certain improvement of the dry-sliding wear behavior of both steels (Figure 11, Figure 12 and Figure 16). For plate 316T, this can be explained by the increase in hardness; for LPBF 316L, the increase in wear resistance can be justified by the elimination of porosity due to plasma-induced surface melting. Notably, as-received and plasma-treated specimens exhibit similarly high friction coefficients, with a large scattering of instantaneous CoF values, indicating that intensive adhesion occurred between the specimen surface and the alumina counter ball. Adhesion forces caused deterioration of the surface layers through a galling mechanism [56,57]; this degradation mechanism is more destructive for the LPBF-printed metal due to the presence of stress concentrators (pores, non-metallic inclusions).

The sliding wear between the counterparts is significantly alleviated when tested in SBF. As a result, large decreases in the CoF scatter and mean value for both types of specimens occurred (compare Figure 16 with Figure 12). SBF provides a lubricating effect and presumably stimulates (through a tribo-corrosion synergism [58]) the formation of protective oxide films (Cr_2_O_3_) [59], which also inhibit adhesion between the contacting surfaces. The lubricating effect is more beneficial for LPBF 316L, since it mitigates adhesion-induced destruction of the as-printed (pore-containing) structure. In wet conditions, the advantage of as-received LPBF 316L over plate 316T in hardness is manifested in a 3-fold advantage of LPBF 316L in terms wear resistance. It can also be highlighted that under SBF conditions, LPBF 316L shows lower CoF than wrought 316T, possibly owing to an additional lubricating effect caused by the presence of Ti-rich oxides in the structure [60].

The SBF solution contains chloride ions responsible for activating electrochemical corrosion. Therefore, relatively poor wear behavior would be expected for the LPBF-printed specimen due to a more pronounced tribo-corrosion synergism in comparison with its wrought counterpart. This assumption is based on the inhomogeneity of the LPBF structure (light contrast/dark contrast pattern of etched structure, Figure 1d–h), which might decrease corrosion resistance, thus increasing total wear. However, as follows from the results, the corrosion issue is not vital for the SBF wear resistance of the LPBF-printed steel, which outperformed the plate steel. Obviously, in this case, the higher hardness of LPBF 316L is more important since it provides less surface deformation. Nevertheless, the elemental distribution between the different areas of the LPBF 316L structure should be further studied in order to understand the effect of selective laser melting on the corrosion resistance of 3D-printed biomedical steel.

Pulsed-plasma modification improved the SBF wear resistance of plate 316T (Figure 11) through surface modification and an increase in concomitant hardness. Improved hardness allows for inhibition of the galling phenomenon, thus decreasing CoF, as seen in Figure 16b. Hence, plasma modification is beneficial for SBF tribological behavior of plate 316T. Conversely, PPT did not improve LPBF 316L wear behavior under SBF sliding (Figure 11 and Figure 16b), since the modified layer has the same cellular structure as the as-printed metal. Moreover, the wet wear resistance of LPBF 316L even decreased. It seems that the beneficial effect of the increase in the surface density of LPBF 316L (due to the elimination of porosity by plasma melting) on the SBF wear performance was counterbalanced by the negative effect of other PPT-induced factors, such as residual stresses in the modified layer or enrichment of the modified layer with non-metallic inclusions (revealed by XRD). Eventually, the wear resistance of plasma-modified plate 316T got much closer to that of LPBF 316 under SBF sliding, as compared to the non-modified state.

The present research shows that LPBF-printed 316L steel presents the same stiffness (elastic modulus) and higher micro-hardness than 316T steel manufactured by conventional technology (rolling). Under dry sliding, LPBF 316L showed inferior wear resistance compared to plate 316T due to porosity and coarse, non-metallic inclusions. Under wet sliding in simulated body fluid, LPBF 316L has an indisputable advantage over plate 316T steel in terms of higher wear resistance and a lower friction coefficient, which makes it preferable for tribological applications in implantology. Pulsed plasma and concomitant surface melting are beneficial (as regards the wear resistance, especially in SBF) mostly for plate steel and less effective for LPBF 316L steel. PPT is beneficial (as regards a low CoF) for both plate 316T and LPBF 316L under SBF-sliding conditions. On the other hand, PPT is beneficial for wrought 316T (as regards a low CoF) under dry sliding, whilst it does not have any significant effect on LPBF 316L.

Poor surface state, porosity and residual stresses may negatively affect the application performance (including fatigue resistance) of additively manufactured metallic parts [61]. Therefore, different post-processing treatments have been proposed to address this challenge [8,14,15,61,62]. They are mostly focused on improving the geometry (e.g., decreasing the surface roughness) by means of abrasive barrel finishing [14,61,63], abrasive flow [64], chemically assisted magnetic abrasive finishing [65], ball burnishing [66,67], etc. The fatigue resistance of LPBF-printed components can also be enhanced by post-processing heat treatment due to residual stress relief and strength anisotropy reduction via removal of the high dislocation density [14,68]. Basically, PPT is considered a hardening technology. However, in the case of LPBF-austenitic stainless steels, PPT is rather effective in modifying the AM metal through porosity elimination and metal purification under plasma-induced melting/crystallization. These PPT attributes could be beneficial for the ability of LBFB stainless steels to successfully withstand fatigue and corrosion. The main advantage of pulsed-plasma treatment is that it can provide a localized modifying effect on the most loaded surface area, thus saving processing cost and time. The assessment of fatigue/corrosive performance of pulsed-plasma-treated LPBF 316L steel is a challenge for future extensive research.

## 5. Conclusions

The microstructure and sliding wear performance of 316 stainless steel manufactured by conventional rolling and laser-based powder bed fusion (LPBF) were investigated in the as-received state and after pulsed-plasma treatment (PPT). The following conclusions can be drawn:

1. LPBF-printed 316L steel features an inhomogeneous etched austenitic structure, presenting “fish-scale” or “drop-like” patterns depending on the observation plane. Additionally, the LPBF-printed 316L structure includes TiC carbides, Ti-based oxides, silicates and pores. LPBF 316L steel has almost the same elastic modulus and nearly double the hardness (mostly attributed to its fine structure and residual stresses), as compared with rolled 316T steel.
2. Under dry sliding, LPBF 316L showed an inferior wear resistance and a slightly higher friction coefficient (CoF), as compared to rolled 316T, which is mainly attributed to the presence of porosity and coarse, non-metallic inclusions in its structure. Sliding in simulated body fluid (SBF) caused a significant decrease in the friction force. Under these conditions, LPBF 316L exhibited lower CoF and three-fold higher wear resistance, as compared to plate 316T, due to a higher hardness and enhanced surface formation of oxide films.
3. PPT resulted in modification of the surface layer to a depth of 22–26 μm due to melting at a heating rate of up to 3.6 × 10^6^ K⋅s^−1^, followed by solidification at a cooling rate of up to 1.6 × 10^6^ K⋅s^−1^. The modified layer presents a cellular structure with refined cells of 0.3–0.7 μm (cross-section) in diameter, normally oriented to the surface. Plasma modification did not affect the elastic modulus of 316 steel. PPT simultaneously caused an increase in the hardness of the rolled steel, due to the transformation of the polygonal structure to a cellular grain structure. However, PPT did not affect the hardness of the LPBF-printed steel.
4. Pulsed-plasma modification moderately improved the dry-sliding wear resistance of both LPBF 316L (due to elimination of porosity during surface melting) and plate 316T (due to an increase in hardness). PPT led to a slight decrease in the CoF of the wrought stainless steel, whereas it did not alter the CoF of the LPBF stainless steel.
5. PPT improved the SBF wear resistance of plate 316T and caused a decrease in the CoF of plate 316T. However, PPT decreased the SBF wear resistance of LPBF 316L, although it led to a decrease in the CoF of the LPBF steel.

## Figures and Tables

**Table 1:** Process (LPBF) and post-process (PPT) parameters.

Process	Equipment	Energy Source	Surface Power Density, Wt⋅m^−2^	Working Atmosphere	Working Parameters
LPBF	ProX DMP 320	Fiber laser (1070 nm, beam diameter: 0.5 mm)	25.5 × 10^8^	Argon (1.5–4.0 bar)	Layer thickness: 30–60μm, printing accuracy: ±50 μm
PPT	Electrothermal axial plasma accelerator	Capacitive energy storage	6 × 10^8^	Air	Charging voltage: 4.5 kV, pulse duration: 1 ms, distance to the target: 50 mm, pulse number: 1.

**Table 2:** EDX chemical composition (wt.%, normalized to 100%) of the non-metallic inclusions in LPBF 316L.

Phase	O	Si	Ti	Nb	Mo	Al	Cr	Fe	Ni
(Ti,Al)O_2_	38.5	-	38.8	-	-	22.7	-	-	-
(Nb,Ti,Mo)C	-	-	5.6	21.2	7.4	-	10.4	23.3	32.1
SiO_2_	47.0	53.0	-	-	-	-	-	-	

## Data Availability

Data sharing is not applicable to this article.
